# Integrated Analysis of the Effects of Cecal Microbiota and Serum Metabolome on Market Weights of Chinese Native Chickens

**DOI:** 10.3390/ani13193034

**Published:** 2023-09-27

**Authors:** Shenghong Yang, Yongxian Yang, Xiaoxia Long, Hui Li, Fuping Zhang, Zhong Wang

**Affiliations:** Key Laboratory of Animal Genetics, Breeding and Reproduction in the Plateau Mountainous Region, Ministry of Education, College of Animal Science, Guizhou University, Guiyang 550025, China; yangshenghong2023@163.com (S.Y.); m18286447263_2@163.com (Y.Y.); xiaoxialong2022@163.com (X.L.); ellenlihui@sina.cn (H.L.); zfu-1010@126.com (F.Z.)

**Keywords:** market weight, cecal microbiota, metabolome, Guizhou yellow chicken, 16S rRNA

## Abstract

**Simple Summary:**

Native chickens generally have the characteristics of low growth performance, which has also become a limiting factor for the breeding of yellow feather broilers. More and more studies have shown that gut microbiota plays an important role in the growth of livestock farming. The Guizhou yellow chicken is a breed of yellow-feathered broiler chicken with excellent meat quality and good flavor currently being cultivated in Guizhou Province, China. In order to explore the role of gut microbiota on the growth performance of native chickens, the Guizhou yellow chicken was taken as a representative, and high-market-weight and low-market-weight chicken groups were established according to their market weights. By integrating microbial 16S rRNA gene sequencing and non-targeted serum metabolome data, five key cecal microbes associated with high body weight in chickens and one key microbe associated with low body weight were identified. In addition, the results also showed that specific gut microbes might positively affect the growth rate of chickens by regulating vitamin and other metabolic pathways. These findings might improve understanding of the role of gut microbiota in chicken growth traits and their underlying metabolic mechanisms.

**Abstract:**

The gut microbiota plays an important role in the physiological activities of the host and affects the formation of important economic traits in livestock farming. The effects of cecal microbiota on chicken weights were investigated using the Guizhou yellow chicken as a model. Experimental cohorts from chickens with high- (HC, *n* = 16) and low-market-weights (LC, *n* = 16) were collected. Microbial 16S rRNA gene sequencing and non-targeted serum metabolome data were integrated to explore the effect and metabolic mechanism of cecal microbiota on market weight. The genera *Lachnoclostridium*, *Alistipes*, *Negativibacillus*, *Sellimonas*, and *Ruminococcus torques* were enriched in the HC group, while *Phascolarctobacterium* was enriched in the LC group (*p* < 0.05). Metabolomic analysis determined that pantothenic acid (vitamin B5), luvangetin (2*H*-1-benzopyran-6-acrylic acid), and menadione (vitamin K3) were significantly higher in HC serum, while beclomethasone dipropionate (a glucocorticoid) and chlorophene (2-benzyl-4-chlorophenol) were present at higher levels in the LC group. The microbes enriched in HC were significantly positively correlated with metabolites, including pantothenic acid and menadione, and negatively correlated with beclomethasone dipropionate and chlorophene. These results indicated that specific cecal bacteria in Guizhou yellow chickens alter the host metabolism and growth performance. This study provides a reference for revealing the mechanism of cecal microbe actions that affect chicken body weight.

## 1. Introduction

Chicken meat is a major protein source throughout the world [1], and the improvement of chicken production performance is necessary to meet increasing demand. Market weight is a key growth trait in chickens, and high market weight can increase the turnover in the chicken production pen and reduce labor costs for farmers [2]. Several factors, such as genetics [3], management [4], and nutrition [5], can affect market weight, thus it can be scientifically altered.

Gut microbes might be involved in the regulation of chicken market weight. For example, the presence of *Lachnospiraceae* in the cecum was found to be related to high growth performance (body weight) for chickens, while the presence of *Escherichia* had the opposite effect [6]. Cecal microbiota possessing *Microbacterium* and *Sphingomonas* in Turpan gamecock progeny × White Leghorn chickens were significantly correlated with high body weight, while *Slackia* was enriched in the ceca of low-market-weight chickens [7]. In addition, high *Lactobacilli* abundance in the chicken jejunum was beneficial to growth, while *Comamonas* enrichment produced a negative outcome on growth rates [8]. The addition of exogenous *Bacillus subtilis* and *Bacillus licheniformis* to chicken feed also promoted growth performance [9,10]. However, the conclusions have not been completely consistent in determining which microbes produce the greatest effect on chicken growth performance.

In spite of numerous studies focused on unraveling the mechanisms for market weight increases, this complex trait has yet to be fully understood [11,12,13]. Since metabolite production might profoundly alter host physiological functions [14,15,16] and thereby affect host phenotypes, such as feed efficiency [17], disease resistance [18], meat quality [19], and gut microbiome can influence host metabolism [15], it is deduced that the influence of gut microbiota on host metabolism might be one of the mechanisms to regulate growth performance of chickens. However, few researches have been found. Only one study proved that cecal microbiota could affect chicken growth performance by regulation of fat metabolism [7]. Unfortunately, there was a limitation in this study, for it focused on only a few specific lipid metabolites. As well known, there are several host metabolites, and the regulatory role of other metabolites remains unclear. Thus, more advanced techniques are necessary for a more comprehensive evaluation.

As a modern omics technology, metabolomics allows high-throughput, multi-dimensional analysis of a large number of metabolites with high sensitivity and can detect very small metabolic changes [20]. Although it has not been directly employed for study on chicken growth performance, it has been applied to fill the information gap between gene and phenotype [21]. With this technology, more comprehensive metabolite information can be obtained. A combination of microbiomes and metabolomics linked gut microbes to the regulation of metabolite production, which is becoming an important means of analyzing complex traits of livestock farming [22,23,24,25]. Based on this, researchers have explored the mechanisms of fat deposition in pigs and milk protein formation in cows. For example, *Prevotella* is a key bacterial genus that affects pig intramuscular fat deposition via the production of lipopolysaccharides, branched-chain amino acids (BCAAs), and arachidonic acids [26]. *Prevotella* abundance in the rumen altered amino acid metabolism, resulting in increased milk protein content. In contrast, the enrichment of methanogens in the rumen was not conducive to increasing the milk protein content [27]. In chicken, multi-omics technology was used for the characterization of *bacillus* spp. probiotics isolated from European broilers to improve their growth performance [28]. Therefore, integrating gut microbiome and metabolomics is helpful for the identification of key microbes related to growth traits and the analysis of their metabolic mechanisms in chickens [29,30].

Guizhou yellow chicken is a breed of yellow-feathered broiler chicken with excellent meat quality and good flavor currently being cultivated in Guizhou Province, China. This synthetic chicken breed was developed by crossing parental breeds (Guizhou Weining chicken as female parent and Golden Plymouth Rock chicken as male parent) [31]. However, compared with commercial broiler breeds such as Ross and Cobb [32], the growth rate of this breed is slow [33]. Furthermore, the factors that influence their growth rates have not yet been fully elucidated.

In this study, it was hypothesized that the specific cecal microbiota might be involved in regulating the market weight of Guizhou yellow chickens by influencing host metabolism. Microbial 16S rDNA gene sequencing and untargeted metabolomics were combined to explore the effect of gut microbiome on the growth performance of Guizhou yellow chickens and to identify key microbes associated with market weight and preliminary explore the possible metabolic mechanisms for improved growth performance. The results of this study could give insight into the analysis of chicken growth performance and provide research references for the discovery of growth-promoting probiotics.

## 2. Materials and Methods

### 2.1. Ethics Statement

The Animal Care and Use Committee at Guizhou University approved this project (approval number: EAE-GZU-2022-T050). All animal works were conducted according to the guidelines for the care and use of experimental animals established by the Ministry and Rural Affairs of the People’s Republic of China.

### 2.2. Experimental Animals and Sample Collection

The animals involved in this study were Guizhou Yellow chickens bred in our laboratory and raised in the chicken farm of Guizhou University (research farm) from June to October 2022. All chickens (*n* = 49, 24 female + 25 male) were hatched on the same day and were raised in cages. Stocking density was as follows: at the age of 0–4 weeks, 4–10 weeks, and 10–18 weeks, 16 chickens (male and female), 8 chickens (male and female), and 1 chicken were kept in each cage, respectively. The ambient feeding temperature was set at 26 °C when the animals were 0–4 weeks old; after that, chickens were transferred to the roller-curtain natural ventilation cooling chicken house without a temperature regulation system and raised at natural room temperature. The temperature was 20–35 °C in June-August and 15–25 °C in September–October. All chickens were fed at 5 AM and 5 PM every day. The light duration was about 16 h per day, and the humidity was 60% to 65%. All chickens were raised in the same environment. The chicken house was cleaned and disinfected regularly according to the sanitary and epidemic prevention requirements of the farm to keep them clean, dry, and ventilated. All the chickens were allowed to eat and drink freely. At different growth stages, the animals were fed brood feed, nursery feed, and growing feed according to different nutritional requirements. The nutritional composition is shown in Table 1. The chickens were weighed at the same time on the morning of the weighing day using an electronic scale every 2 weeks (±5 g). At 18 weeks of age, 8 roosters and 8 hens with the highest body weight were selected into the high-market-weight group (HC, *n* = 16), and 8 roosters and 8 hens with the lowest body weight were selected into the low-market-weight group (LC, *n* = 16). Namely, in each group, 16 replicates were involved.

**Figure 1:**
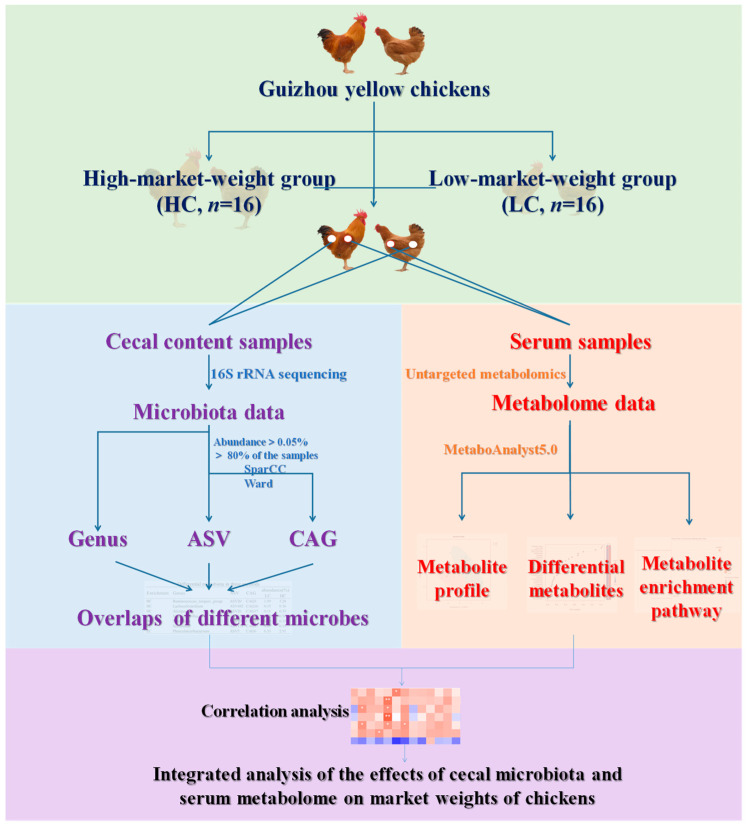
Experimental flow chart. The experimental cohort comprises 49 healthy Guizhou yellow chickens; 8 roosters and 8 hens that possessed the highest body weights in the group constituted the high-market-weight group (HC, *n* = 16), and a similar group was selected for the low-market-weight group (LC, *n* = 16). Cecal samples were collected and subjected to 16S rRNA sequencing to infer microbial profiles. Concurrent blood samples were collected to perform untargeted metabolomics detection. Cecal and serum metabolites were identified by statistical analysis.

No antibiotics were used within one month before slaughter. At the end of the experiment (18-week-old), serum samples and cecal contents were collected from all 32 chickens (16 in the high-market-weight group and 16 in the low-market-weight group). Whole blood was obtained from wing veins for serum separation before the chickens were euthanized by CO_2_ asphyxiation [34], and then ~2 g cecal content was collected at the same position as their cecum. Serum samples and cecal content were immediately put into liquid nitrogen for quick freezing after isolation or collection and were stored at −80 °C for further analyses. Thirty-two serum samples (16 from HC, 16 from LC) for untargeted metabolomics and 32 cecal content samples (16 from HC, 16 from LC) for 16S rDNA gene sequencing were performed at Shanghai Applied Protein Technology (Aptbio, Shanghai, China) and Shanghai Majorbio Biopharm Technology (Majorbio, Shanghai, China), respectively. The experimental flow chart is shown in Figure 1.

### 2.3. Sequence Splicing and ASV Annotation

Amplicon sequencing was conducted using an Illumina MiSeq platform (Illumina, San Diego, CA, USA). The reads were first filtered and assembled into tags according to overlap relationships between the paired-end reads. Tags were then clustered into amplified sequence variants (ASVs) [35]. Data was optimized using the DADA2 to obtain the representative ASV sequence and abundance information [36]. Representative sequences of each ASV were annotated using the Silva database (https://www.arb-silva.de/) (accessed on 22 February 2023) with a taxonomic confidence level of 0.7 [37].

### 2.4. Extraction of Serum Samples

Serum samples were thawed at 4 °C, and an appropriate amount was added to pre-cooled a methanol/acetonitrile/water solution (2:2:1, *v*/*v*) and vortexed and ultrasonicated at low temperature for 30 min and kept at −20 °C for 10 min. Samples were then centrifuged at 14,000× *g* at 4 °C for 20 min. Supernatants were transferred into clean tubes and dried under vacuum, and the residue was suspended in 100 μL 50% acetonitrile for UPLC-QTOF/MS analysis [38].

### 2.5. Chromatography-Mass Spectrometry Analysis

#### 2.5.1. Chromatographic Conditions

Sample compounds were separated using an Agilent (Agilent Technologies, Santa Clara, CA, USA) 1290 Infinity LC ultra-high performance liquid chromatography system using a hydrophilic interaction (HILIC) column using the mobile phases as follows: A (25 mM ammonium acetate and 25 mM ammonia in H_2_O) and B (acetonitrile). The gradient elution procedure is as follows: 0–0.5 min, 95% B; 0.5–7 min, B 95 to 65%; 7–8 min, B 65 to 40%; 8–9 min, B 40%; 9–9.1 min, B 40 to 95%; 9.1–12 min, B 95%. During this process, the proportion of mobile phase A changed accordingly. Samples were placed in an autosampler tray at 4 °C. QC samples were inserted into the sample queues to monitor and evaluate the stability of the system and the reliability of experimental data [39].

#### 2.5.2. Data Processing

Proteo Wizard MS Convert was used to convert the raw MS data to MzXML files before importing it into freely available XCMS software (v 1.52.0) [40]. For peak picking, the following parameters were used: centWave *m*/*z* = 10 ppm, peakwidth = c (10, 60), prefilter = c (10, 100). For peak grouping, bw = 5, mzwid = 0.025, minfrac = 0.5 were used. Isotopes and adducts were annotated by CAMERA (Collection of Algorithms of MEtabolite pRofile Annotation) [41]. In the extracted ion features, only the variables with more than 50% of the nonzero measurement values in at least one group were kept [41]. Compound identification of metabolites was conducted by comparing accuracy *m*/*z* value (<10 ppm) and MS/MS spectra with an in-house database established with available authentic standards [42].

### 2.6. Statistical Analysis

#### 2.6.1. Analysis of Microbiota Diversity and Composition Differences

Analysis for taxonomic clusters utilized the ASVs, and quality control was carried out under conditions of relative abundance >0.05% and detection of ASVs in >80% of the samples [43]. ASV α-diversity with Shannon, Simpson, Chao1, Faith’s phylogenetic diversity (PD), and ACE indices were calculated using Mothur software v 1.31.2 [44]. The Wilcoxon rank sum test was used to compare α-diversity differences between the two groups. Principal coordinates analysis (PCoA) [45] was performed to evaluate the discrepancy of the phylogenetic compositions of cecal microbiota between HC and LC. Linear discriminant analysis with difference contribution analysis (LEfSe) was performed with LDA > 2 and *p* < 0.05 as thresholds to identify bacterial composition differences between the two groups [46].

#### 2.6.2. Construction of Cecal Microbial Co-Abundance Groups

The quality-controlled ASVs were used to construct co-abundance groups (CAGs) of cecal microbiota. The correlation matrix between ASVs was calculated based on the SparCC algorithm [47] using the SpiecEasi package in the R [48]. Paired ASVs with a correlation coefficient >0.5 were used for further analyses. The correlation coefficient values were converted into correlation distances (1-correlation coefficient), and ASVs were clustered into CAGs based on the Ward algorithm with the Vegan package in the R [49]. Cytoscape v 3.9.1 [50] was used for the visualization of cecal microbiota CAGs.

#### 2.6.3. Serum Metabolomics Analysis

After sum-normalization, the online analysis platform MetaboAnalyst 5.0 (https://www.metaboanalyst.ca/) (accessed on 14 March 2023) was employed to analyze the processed data by multivariate data analysis, including Pareto-scaled principal component analysis (PCA) and orthogonal partial least-squares discriminant analysis (OPLS-DA) [51]. The robustness of the model was evaluated using 7-fold cross-validation and response permutation testing. The variable importance in the projection (VIP) value of each variable in the OPLS-DA model was calculated to indicate its contribution to the classification [52]. In order to determine the significance of differences between two groups of independent samples, the Student’s t-test was applied. VIP > 1 and *p* < 0.05 were used to screen significantly changed metabolites. Pearson’s correlation analysis was performed to determine the correlation between two variables [49].

## 3. Results

### 3.1. Growth Performance of the Study Chickens

**Figure 2:**
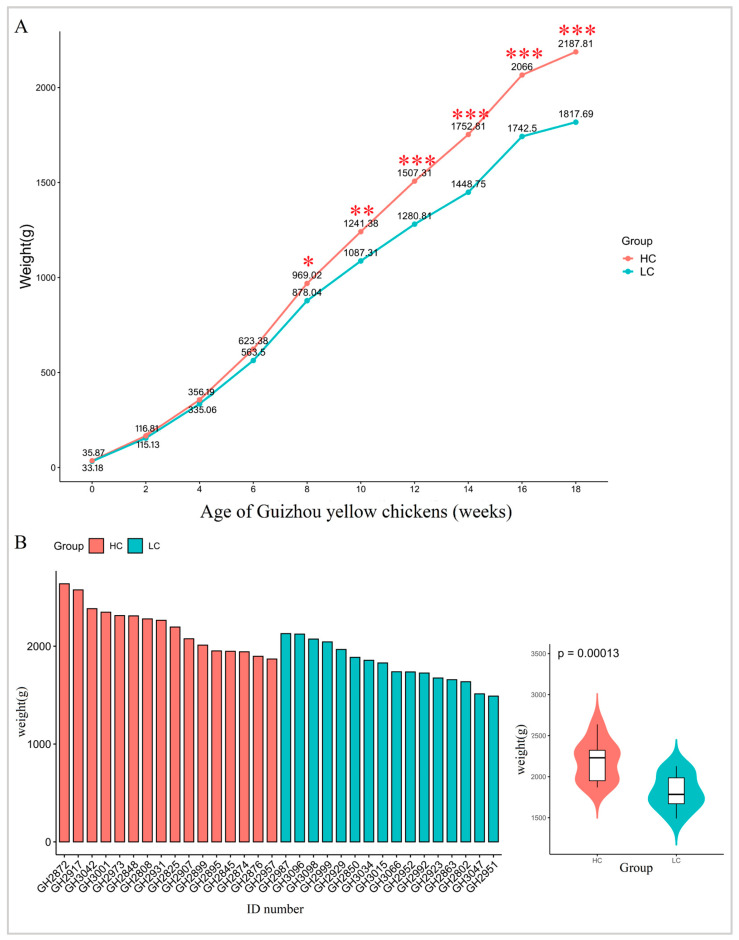
Body weights of Guizhou yellow chickens. (**A**) Changes in body weight of ages 0 to 18 weeks. * *p* < 0.05, ** *p* < 0.01, *** *p* < 0.001. (**B**) Body weight distribution of experimental chickens at 18 weeks of age. HC, high-market-weight chicken group (*n* = 16). LC, low-market-weight chicken group (*n* = 16).

The weight gain trends for the HC and LC groups of chickens were consistent from 0 to 18 weeks of age when the animals were provided with identical environments and feed and water access. There was no significant difference in body weights between the two groups from 0 to 6 weeks of age. In contrast, from week 8 onwards, body weight differences reached the level of statistical significance (*p* < 0.05) (Figure 2A). By 18 weeks of age, the body weights for HC (2187.81 ± 232.58 g) were significantly (*p* < 0.01) greater than the LC group (1817.69 ± 199.30 g) (Figure 2B and Table 2). 

### 3.2. Cecal Microbial Diversity in High- versus Low-Market-Weight Chickens

**Figure 3:**
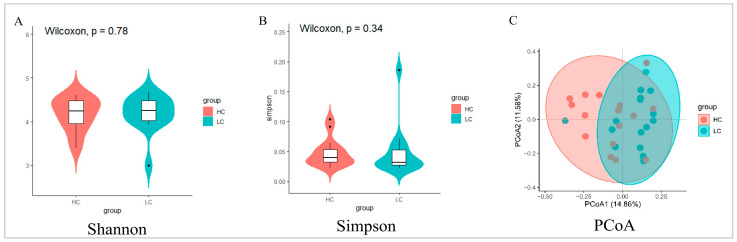
Differences of cecal microbiota diversity between high- and low-market-weight chickens. Comparison of α-diversity in cecal microbiota between HC and LC using the (**A**) Shannon and (**B**) Simpson indices. (**C**) PCoA of cecal microbiota from HC and LC. HC, high-market-weight chicken group (*n* = 16). LC, low-market-weight chicken group (*n* = 16).

A total of 32 microbial DNA samples from HC and LC chickens were used for 16S rDNA gene sequencing, 1,535,941 clean reads were generated after QC, and 1955 ASVs were identified for all samples (Table S1). The Shannon and Simpson of α-diversity indices between the two groups were of no significant differences (Figure 3A,B). PCoA analysis showed a lack of significant differences in β-diversity of the HC and LC cecal microbiota, although a certain aggregation effect was evident (Figure 3C).

### 3.3. Bacteria Differentially Abundant in HC versus LC

**Figure 4:**
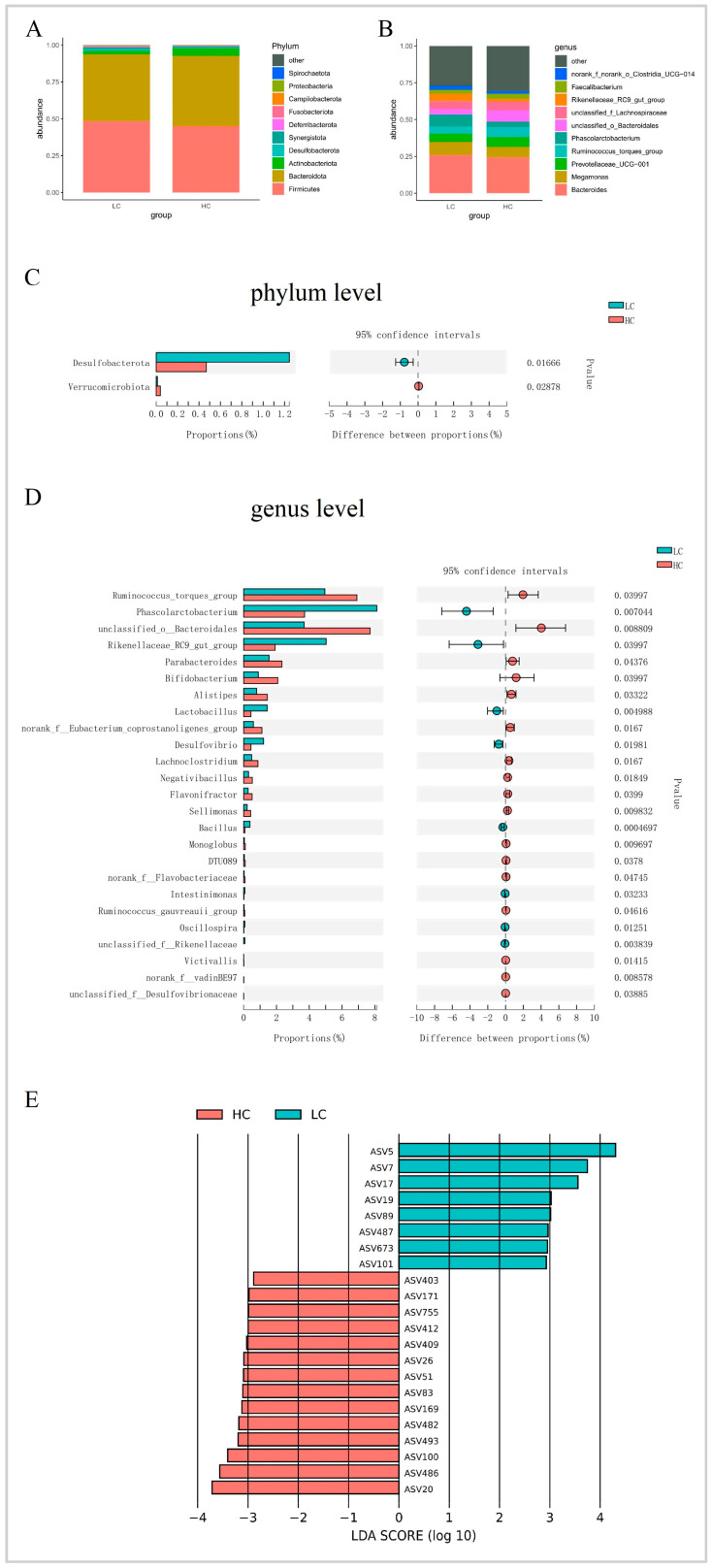
Differences of cecal microbiota between high- and low-market-weight chickens. Comparison of cecal microbiota composition between HC/LC at the (**A**) phylum and (**B**) genus levels (relative abundance > 0.05%). Cecal microbiota showing different abundances at the (**C**) phylum and (**D**) genus levels between HC and LC (relative abundance > 0.05%). (**E**) Differential ASVs identified by LEfSe analysis between HC and LC (relative abundance > 0.05%). HC, high-market-weight chicken group (*n* = 16). LC, low-market-weight chicken group (*n* = 16).

At the phylum level and based on relative abundance, the cecal microbiota of the Guizhou yellow chicken was primarily composed of Firmicutes (46.88%), Bacteroidota (46.28%), Actinobacteriota (3.91%), Desulfobacterota (0.86%), and Synergistota (0.85%) (Figure 4A and Table S2). Comparison based on abundance for HC and LC indicated that Verrucomicrobiota were present at significantly higher levels in HC (Wilcoxon test, *p* = 0.017) while Desulfobacterota were significantly (*p* = 0.029) higher in the LC group (Figure 4C and Table S3). At the genus level, the dominant bacteria were *Bacteroides* (25.15%), *Megamonas* (8.05%), *Prevotellaceae UCG-001* (5.98%), *Ruminococcus torques group* (5.93%) and *Phascolarctobacterium* (5.92%) (Figure 4B). A comparison by abundance for HC and LC identified 17 genera. In the HC group, the most abundant were the *Ruminococcus torques group*, *unclassified Bacteroidales*, *Parabacteroides*, *Bifidobacterium*, and *Alistipes.* The LC group was enriched in eight genera, including *Phascolarctobacterium*, *Rikenellaceae RC9 gut group*, *Lactobacillus*, *Desulfovibrio*, and *Bacillus* (Figure 4D).

Characteristics of the cecal microbiota for the LC and HC groups were further explored at the ASV level using LEfSe analysis. Twenty-two out of two hundred and thirty-three (22/223) ASVs displayed differential abundances, among which eight ASVs were significantly enriched in LC, and fourteen were significantly enriched in HC (LDA > 2, *p* < 0.05). Again, *Ruminococcus torques group* (ASV20), *Alistipes* (ASV26, ASV169), *Lachnoclostridium* (ASV482) were enriched in HC, and *Phascolarctobacterium* (ASV5, ASV7), *Lactobacillus* (ASV17, ASV19) and *Bacillus* (ASV487) were enriched in LC and also displayed significant differences at the genus level (Figure 4E, Table 3).

### 3.4. Identification of Co-Abundance Groups (CAG) Associated with Body Weight

**Figure 5:**
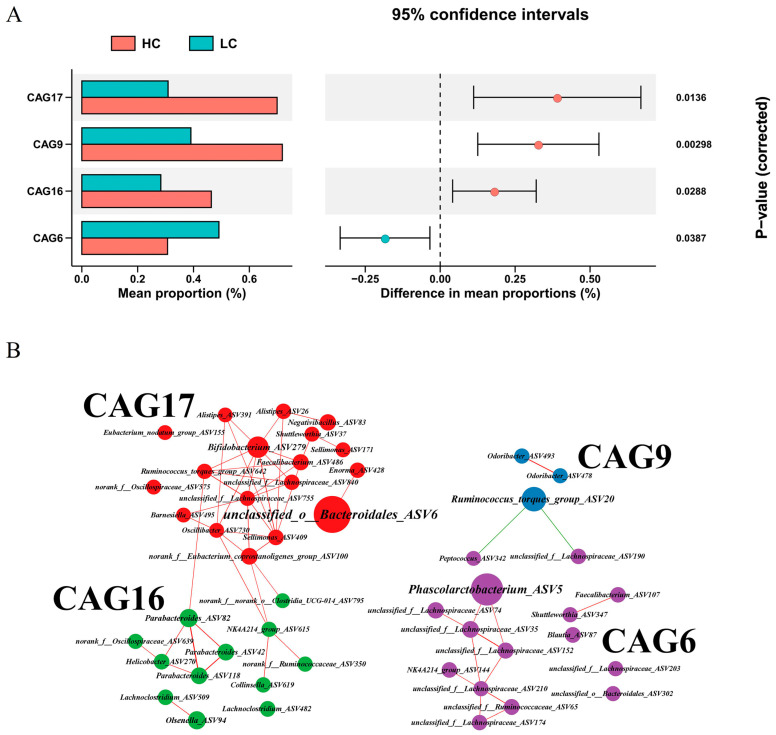
Co-abundance groups (CAG) of cecal microbiota associated with high- and low-market-weight chickens. (**A**) Association of CAGs with HC or LC. CAG6 was enriched in the cecal microbiota of LC, and CAG9, CAG16, and CAG17 were enriched in the cecum of HC. (**B**) Comparison of mean relative abundance in four differential CAGs between HC and LC. HC, high-market-weight chicken group (*n* = 16). LC, low-market-weight chicken group (*n* = 16).

Another goal of this study was to explore the gut microbiota clusters associated with chicken body weight [53]. The identified 223 ASVs were clustered into 20 CAGs, and the average relative abundance of each CAG was compared between groups using the Wilcoxon test. Four CAGs were identified that significantly differed between the two groups: CAG6, CAG9, CAG16, and CAG17 (Figure 5A). CAG6 was enriched in LC and contained 22 ASVs, and *Phascolarctobacterium* ASV5 was the most enriched in LC. This suggested that CAG6 with *Phascolarctobacterium* as the core has a potentially negative effect on growth performance. In contrast, CAG17 was enriched in HC and included *Ruminococcus torques group* ASV642, *Bifidobacterium* ASV279, *Alistipes* ASV26, *Alistipes* ASV239, and others. In addition, CAG9 was centered on *Ruminococcus torques group* ASV20, while CAG16 contained bacteria such as *Parabacteroides* that were enriched in HC at the genus level (Figure 5B and Table S4).

### 3.5. Differential Serum Metabolites between HC and LC

**Figure 6:**
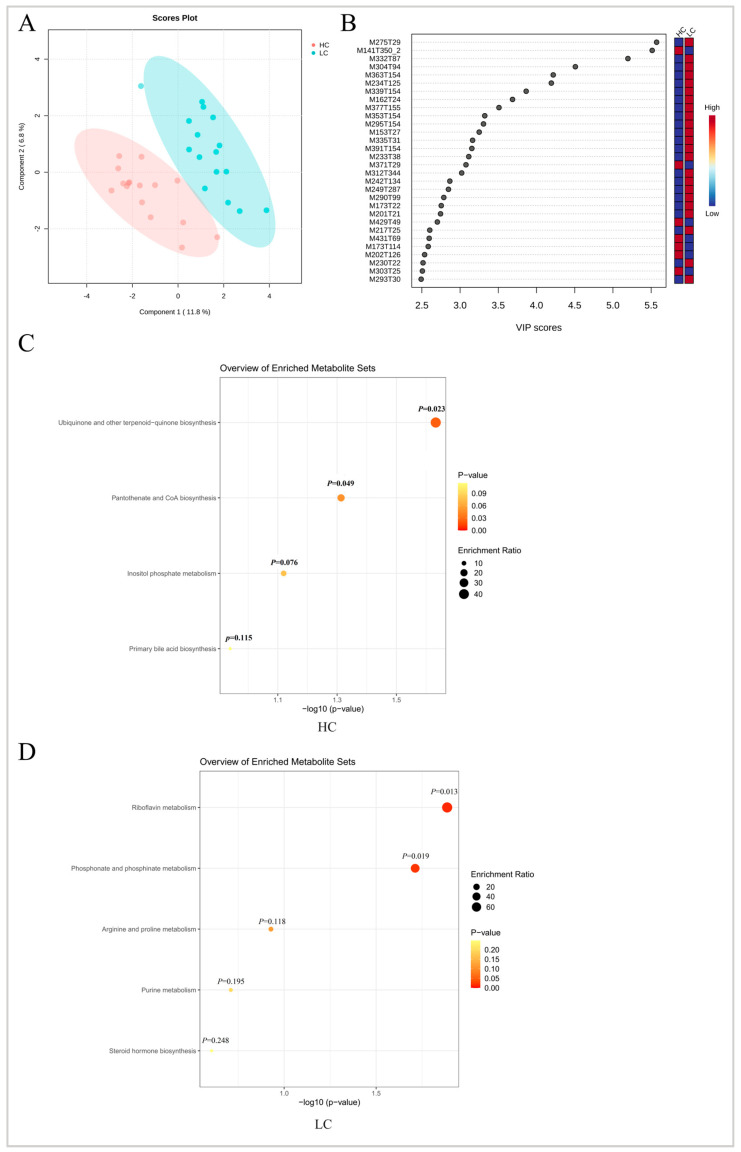
Identification of the serum metabolites enriched in high- and low-market-weight chickens. (**A**) Partial least squares discriminant analysis (PLS-DA) of untargeted serum metabolome data from HC and LC. The pink and blue markers represent HC and LC, respectively. (**B**) Variable importance in projection (VIP > 1) scores for the top serum metabolites contributing to variation in metabolic profiles of HC and LC (Only showed the top 30, the annotation of metabolites are showed in Table 4). Metabolic pathways enriched in (**C**) HC and (**D**) LC. HC, high-market-weight chicken group (*n* = 16). LC, low-market-weight chicken group (*n* = 16).

Untargeted metabolome assays were performed on serum samples from all 32 chickens, and a total of 766 annotated serum metabolites were detected, including 494 positive ion metabolites and 272 negative ion metabolites. Metabolomics data analysis was performed on the MetaboAnalyst 5.0 online platform, and the PLS-DA results indicated that the metabolites of HC and LC were significantly separated (Figure 6A). The metabolites annotated by the positive and negative ion modes were combined, and the differential metabolites were compared at a threshold of VIP > 1, *p* < 0.05. There were 44 metabolites showing significant differences between the two groups, including 13 metabolites such as pantothenic acid, luvangetin (2*H*-1-benzopyran-6-acrylic acid), metyrapone (methyl-1,2-di-pyridyl-1-propanone) and sempervirine (16,17,18,19-tetrahydroyohimban) that were at higher levels in group HC. In LC, we identified 31 significant metabolites including phosphocreatine, glufosinate (2-amino-4-[hydroxy(methyl)phosphoryl] butanoic acid), hesperetin ((2S)-5,7-dihydroxy-2-(3-hydroxy-4-methoxyphenyl)-2,3-dihydrochromen-4-one and chlorophene (2-benzyl-4-chlorophenol) (Figure 6B, Table 4). Pathway enrichment analyses were then performed with these differential metabolites. In the HC group, four metabolic pathways were enriched, including ubiquinone and other terpenoid-quinone biosynthesis, pantothenate, and CoA biosynthesis (Figure 6C). In the LC group, five metabolic pathways were enriched, including riboflavin metabolism, phosphonate, and phosphinate metabolism (Figure 6D).

### 3.6. Correlation Analysis Reveals Relationships between the Cecal Microbiota and Serum Metabolites

**Figure 7:**
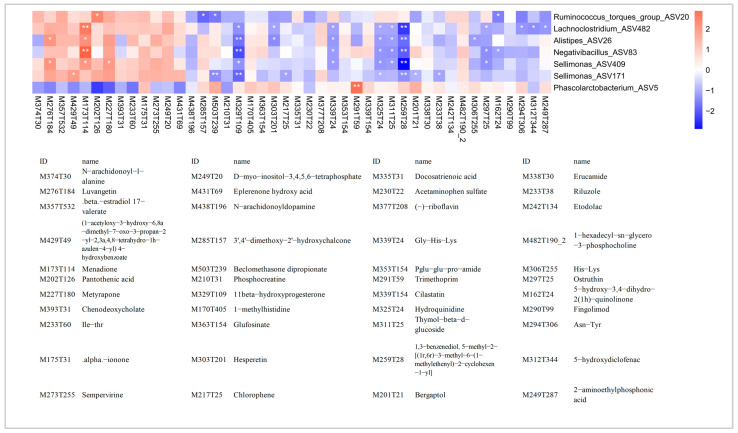
Correlation between differential serum metabolites and weight-related cecal microbes. Red indicates a positive correlation, while blue indicates a negative correlation. * *p* < 0.05, ** *p* < 0.01.

The overlaps of differential bacteria at the ASV, genus, and CAG levels were integrated. The *Ruminococcus torques group*, *Lachnoclostridium*, *Alistipes*, *Negativibacillus*, and *Sellimonas* were enriched in HC, and *Phascolarctobacterium* was enriched in LC in all analyses at three levels (Table S5). Spearman’s rank correlation analysis was then performed between these bacteria and differential metabolites, and the *Ruminococcus torques group* enriched in HC was significantly positively correlated with pantothenic acid (*p* = 0.029, *r* = 0.386). In addition, menadione was positively correlated with numerous bacteria enriched in HC. These included *Lachnoclostridium* ASV482, *Alistipes* ASV26, *Negativibacillus* ASV83, and *Sellimonas* ASV409, suggesting these bacteria might promote the synthesis of menadione and have beneficial effects on the growth performance of the chickens. The detailed relationships between weight-related bacteria and differential metabolites are shown in Figure 7.

## 4. Discussion

Growth performance is an important trait for chickens, and gut microbiota have been proven to play an important role in the life activities of hosts. However, clear correlations between the gut microbes and chicken growth performance have not been fully revealed. In this study, multi-omics was used to describe the cecal microbiota composition and serum metabolite differences between high- and low-market-weight Guizhou Yellow chicken. The results showed that *Lachnoclostridium*, *Alistipes*, *Negativibacillus*, *Sellimonas*, and *Ruminococcus torques* are beneficial for chicken growth by regulating metabolites such as pantothenic acid and menadione, while *Phascolarctobacterium* might inhibit the growth of chicken.

The composition of the gut microbiota for individual animals also varies by location within the animal, and the diversity and abundance of microbiota in the cecum is the highest. Therefore, cecal microbiota was taken as representative gut microbiota in this study [54,55,56]. Diversity is an important index to evaluate the community structure of microbiota [57]. It has been proved that the diversity of gut microbiota is negatively correlated with weight gain [58]. Decreased diversity of gut microbiota has been linked to inflammatory diseases, which tend to result in fat deposition and increased body weight [26]. However, results from other studies found no significant difference between the cecal microbiota diversity of high-market-weight and low-market-weight chickens [7], which is consistent with the observation in this study. These results are controversial as of now; more studies are therefore needed.

It was found in this study that at the genus level, *Lachnoclostridium*, *Alistipes*, *Negativibacillus*, *Sellimonas*, and *Ruminococcus torques* were enriched in the HC group, while *Phascolarctobacterium* was enriched in the LC group. These specific cecal microbes might be an important factor influencing the body weight of chickens. *Alistipes* and *Lachnoclostridium* are considered to be important producers of short-chain fatty acids, including butyric and acetic acids. It has been proved that a reduction in *Alistipes* abundance is linked to a reduction in the levels of short-chain fatty acids [59,60], and *Alistipes finegoldii* was specifically proved to promote the growth of broiler chickens [60,61,62]. In mice, a reduced *Lachnoclostridium* abundance was associated with decreased body weight [63]. The reason might be that in addition to influencing the production of short-chain fatty acids, *Lachnoclostridium* is also linked to host nutrient absorption, and its reduced abundance will lead to the downregulation of functional pathways such as protein processing and nutrient transport in the host [59,64,65]. *Negativibacillus* is a Gram-negative Firmicute, and its abundance in the mouse gut was positively correlated with body weight gain [66]. *Sellimonas* is an obligate anaerobic, non-motile Gram-positive first isolated from human feces in 2016 [67]. In addition, *Ruminococcus torques* were found to be enriched in chickens with high body weight. It has not been studied much since *Ruminococcus torques* was first described. However, it is often involved in studies that link human microbiota to disease states. For instance, it has decreased abundance in patients with Crohn’s Disease in comparison to healthy individuals [68]. In contrast, its abundance increased in children with late-onset autism [69] and those with autism spectrum disorders and gastrointestinal disorders [70]. Therefore, the role of *Ruminococcus torques* in contribution to health or disease is still an open question [71].

Previous studies have found that there are several mechanisms explaining how gut microbiota affect the growth of the host, such as being involved in vitamin synthesis [72,73], dietary fiber degradation [74], inflammatory induction [33], and lipid metabolism [7]. As well known, vitamins play a catalytic role in promoting nutrient synthesis, thereby controlling metabolism and affecting the performance and health of poultry [75]. Humans and animals cannot synthesize most vitamins by themselves and must obtain them from their diet or rely on gut microbiota to synthesize them [76]. Probiotics such as *Bifidobacterium* and *lactobacillus* could synthesize a variety of vitamins necessary for human growth and development, such as vitamins B [72], vitamin K [77], and vitamin D [73]. In this study, several vitamin metabolites related to body weight were identified by serum metabolome analyses. For example, pantothenate acid and menadione were enriched in the serum of high-market-weight chickens, while riboflavin was enriched in the serum of low-market-weight chickens, which highlights the importance of vitamin metabolism for the regulation of growth traits in chickens.

Pantothenic acid, also known as vitamin B5, is an indispensable essential nutrient that can be converted into Coenzyme A (CoA) and Acyl carrier protein (ACP) in living organisms, both of which are enzyme cofactors necessary for key pathways of metabolism and energy production in all living cells [78]. Pantothenic acid is involved in the metabolism of sugar, fat, and protein in both humans and animals [79]. In this study, a higher concentration of pantothenic acid was found in the serum of high-market-weight chickens. This indicates that pantothenic acid could promote the growth and development of broilers. Early studies showed that deficiency of pantothenic acid caused symptoms such as loss of appetite, growth retardation, and wasting in sick chickens [80,81]. Lacking pantothenic acid in the diet for chicks could result in a decrease in protein, fat, and energy stores, and the addition of pantothenic acid in the diet could not only enhance the activity of intestinal digestive enzymes but also promote the digestion and absorption of nutrients in the diet, thus promoting the growth of animal body [82]. In addition, it was found in this study that *Ruminococcus torques* were enriched in high-market-weight chickens and were significantly positively correlated with pantothenic acid, suggesting that *Ruminococcus torques* might promote the generation of pantothenic acid and, in turn, promote the growth of chickens.

In this study, a significantly higher concentration of menadione (VK_3_) in high-market-weight chickens was found. It is deduced that the beneficial effects of menadione on bone development and its strong antioxidant effect might be two of the reasons for the chickens’ higher market weight. Menadione is a fully reduced form of vitamin K. In addition to its well-known anticoagulant effects, vitamin K also plays an important role in bone formation and remodeling [75]. It has been proved that supplementation of vitamin K in the diets of starter(8 mg/kg feed) and grower (2 mg/kg feed) broilers promoted the carboxylation of osteocalcin and improved the hydroxyapatite binding ability of serum osteocalcin, in turn, improved the bone quality [83]. What’s more, menadione has a strong antioxidant effect [84]. Oxidative stress adversely affects the growth performance of animals because it can cause disorders of chicken gastrointestinal peristalsis, which tend to result in enteritis and diarrhea, damage of intestinal villi, in turn, lead to poor absorption of nutrients, and reduce the nutrient absorption capacity of chickens [85]. The chickens involved in this study were raised from June to October when the temperature was up to 33 °C; natural ventilation might not cool the chicken house effectively, which might cause heat stress in chickens. Some chickens with poor tolerance to heat stress might grow slower, while the high-market-weight chickens may alleviate heat stress by increasing the synthesis of menadione.

It was early discovered that menadione could be produced by many bacteria, such as *Bacillus cereus*, *B. mycoides*, *B. subtilis*, *Chromobacterium prodigiosus*, *Escherichia coli*, *Mycobacterium tuberculosis*, *Sarcina lutea*, and *Staphylococcus aureus* [86], and human gut microbiota (e.g., *Bacteroides* and *Prevotella*) might participate the synthesis of menadione [87]. Interestingly, menadione was found to be enriched in high-market-weight chickens and was significantly associated with a variety of high-market-weight-associated bacteria, including *Lachnoclostridium*, *Alistipes*, *Negativibacillus*, and *Sellimonas*. This suggests that these microbiotas might promote the synthesis of menadione and thus affect the growth of chickens. However, its mechanism needs further study.

In the present study, *Phascolarctobacterium* was found to be a key microbe that was enriched in the cecum of low-weight chickens. *Phascolarctobacterium* is an obligate anaerobic originally isolated from koala feces [88]. Another study also found that *Phascolarctobacterium* abundance was higher in low-feed conversion chickens and was related to a low nutrient absorption capacity of the host. However, the study did not elucidate a detailed mechanism [89]. An additional study found that the abundance of *Phascolarctobacterium* in the gut increases when chickens are exposed to high temperatures for extended periods, and this exposure also results in elevated levels of heat shock proteins and related inflammatory gene expression [90]. Heat stress altered the structure and function of enzymes in the chicken body, reduced the pH of the blood, and caused metabolic acidosis. These were negative influences on chicken growth [91,92], and intestinal inflammation also decreases nutrient absorption, causing body weight to drop [93,94,95]. The presence of *Phascolarctobacterium* was correlated with the induction of inflammation under the action of heat stress and other harmful factors in chickens. These results are consistent with those of this study, where *Phascolarctobacterium* was enriched in the cecum of low-market-weight chickens. As mentioned above, elevated serum levels of some vitamin-related metabolites in high-market-weight chickens might contribute to the relief of heat stress.

In addition, it is worth noting that in this study, 11 beta-hydroxyprogesterone showed higher concentration in the serum of low-market-weight chickens. 11 beta-hydroxyprogesterone is a naturally occurring, endogenous steroid and derivative of progesterone [96]. This might be because at the age of 18 weeks, the low-market-weight chickens were already sexually mature, and the follicular granulosic cells released related sex hormones, making them lay eggs. The onset of sexual maturity of chickens might slow down their growth because the nutrients would be utilized for reproduction instead of growth [97].

## 5. Conclusions

In this study, 16S rRNA gene sequencing with untargeted serum metabolomics was combined to identify cecal microbes associated with body weight in chickens and to preliminarily identify the metabolic mechanism of cecal microbiota affecting growth performance. The effect of cecal microbiota composition on chicken growth performance and its potential mechanism was investigated. The genera *Lachnoclostridium*, *Alistipes*, *Negativibacillus*, *Sellimonas*, *Sellimonas*, and *Ruminococcus torques group* had beneficial effects on the growth performance of chickens, while *Phascolarctobacterium* correlated with low growth performance. A correlation analysis revealed links between specific gut microbiota and serum metabolites. In conclusion, certain cecal microbiotas might increase the market weight of chickens by promoting the utilization of pantothenic acid and menadione.

The results of this study provide new insights into the role of gut microbiota in regulating the growth performance of chickens and also lay the foundation for the subsequent development of chicken growth-promoting probiotics or prebiotic-related products. However, there are some limitations that should be addressed or avoided in subsequent research. Firstly, this research was a small sample, single-center, cross-sectional study. Secondly, only the cecal microbiota and serum metabolites at the time of slaughter were analyzed, and samples were not collected at different growth stages. Therefore, the causal mechanism between gut microbiota and growth traits needs to be further studied through larger sample sizes, using a multi-center design and applying the innovative research techniques of integrated omics technology.

## Figures and Tables

**Table 1:** Composition and nutrient level (air-dried basis).

Item	0–6 Weeks	6–18 Weeks
Ingredients, %		
Corn	56.30	58.62
Soybean meal	18.52	25.00
Rapeseed meal	10.00	0.00
Corn gluten meal	6.33	3.05
Wheat bran	2.94	5.63
Soybean oil	1.63	3.34
Limestone	1.18	1.18
Phytase	0.04	0.00
Choline chloride	0.15	0.00
Methionine	0.15	0.10
Lysine	0.22	0.32
NaCl	0.15	0.15
CaHPO_4_	1.89	1.61
Premix ^1^	0.05	1.00
Total	100.00	100.00
Nutrients ^2^, %		
CP	21.18	19.05
ME, MJ/kg	12.12	12.56
Ca	1.0	0.90
AP	0.45	0.40
Met + Cys	0.90	0.72
Lys	1.06	0.90

^1^ Provides per kg of diet: 0–6 weeks: Vitamin A, 6000 IU; Vitamin B_1_, 2.0 mg; Vitamin B_2_, 4.0 mg; Vitamin B_5_, 42 mg; Vitamin B_6_, 4.0 mg; Vitamin B_12_, 0.01 g; Vitamin D_3_, 2000 IU; Vitamin E 30 IU; Vitamin K_3_, 1.8 mg; Calcium pantothen-ate, 10.0 mg; Biotin, 0.15 mg; Folic acid, 0.85 mg; Fe, 80 mg; Cu, 8.0 mg; Mn, 80 mg; Zn, 65 mg; I, 0.50 mg; Se, 0.25 mg. 6–18 weeks: Vitamin A, 10,000 IU; Vitamin B_1_, 2.50 mg; VB_2_, 7.5 mg; Vitamin B_6_, 3 mg; Vitamin D, 2000 IU; Vitamin E, 25 mg; Vitamin K, 2.8 mg; Nicotinamide 40 mg; Calcium pantothenate, 25 mg; Biotin 0.20 mg; Folic acid 1.5 mg; Vitamin B_12_, 0.015 mg, Fe, 80 mg; Cu, 8 mg, Mn, 100 mg; Zn, 60 mg; I, 0.35 mg; Se 0.3 mg. ^2^ CP was a measured value, while the others were calculated values.

**Table 2:** Body weights of Guizhou yellow chickens at the age of 18 weeks.

ID	Group	Sex	Weights (g)										Average Weights (g)			
			0 Week	2 Weeks	4 Weeks	6 Weeks	8 Weeks	10 Weeks	12 Weeks	14 Weeks	16 Weeks	18 Weeks	Age	LC	HC	*p*-Value (Wilcoxon Test)
GH2951	LC	F	35.2	154.5	337	512	844.4	1095	1173	1327	1464	1490	0 week	33.18	35.87	0.665
GH3047	LC	F	29	130.4	251	553	808.9	1005	1116	1227	1460	1513				
GH2802	LC	F	37.5	94.8	190	387	559.5	764	1060	1207	1551	1637				
GH2863	LC	F	36.1	164.7	364	666	922.9	961	1273	1401	1603	1658	2 weeks	155.13	160.79	0.616
GH2923	LC	F	35.1	147.7	318	368	821.8	1014	1231	1334	1600	1675				
GH2992	LC	F	32.8	138	310	506	794.8	1028	1221	1427	1646	1726				
GH2952	LC	F	33.3	149	287	507	776.4	1045	1275	1465	1702	1737	4 weeks	335.06	345.30	0.220
GH3066	LC	F	31.7	140.9	311	582	953.7	1163	1339	1512	1622	1739				
GH3015	LC	M	42.2	166.6	421	743	1055.5	1212	1404	1493	1778	1829				
GH3034	LC	M	31.9	184.2	352	391	870	1181	1273	1415	1756	1856	6 weeks	563.50	592.53	0.132
GH2850	LC	M	31.2	164.8	365	543	906.3	1246	1349	1521	1861	1886				
GH2929	LC	M	36.7	178.8	345	690	952.8	1182	1315	1539	1877	1967				
GH2999	LC	M	32.5	182.5	342	584	933.9	1169	1306	1478	1915	2044	8 weeks	878.04	922.15	0.023
GH3098	LC	M	29.5	163	376	622	903.9	1035	1321	1573	2016	2073				
GH3096	LC	M	28.3	159.7	401	642	937.7	1213	1521	1687	2037	2124				
GH2987	LC	M	27.9	162.5	391	720	1006.2	1084	1316	1574	1992	2129	10 weeks	1087.31	1162.01	0.005
GH2957	HC	F	34.6	135.8	319	509	823.5	961	1276	1498	1755	1869				
GH2876	HC	F	60.6	147.8	321	608	893.3	1098	1276	1477	1840	1897				
GH2874	HC	F	32.5	175.5	374	616	946.9	1252	1417	1574	1851	1943	12 weeks	1280.81	1390.63	<0.001
GH2845	HC	F	32.1	283.1	352	649	934	1134	1506	1663	1884	1948				
GH2895	HC	F	27.8	160.7	342	548	900.1	1164	1345	1561	1841	1952				
GH2899	HC	F	55.9	142.7	219	443	719.9	1016	1243	1475	1891	2011	14 weeks	1448.75	1596.17	<0.001
GH2907	HC	F	32.8	179.6	395	598	974.9	1245	1545	1773	2003	2076				
GH2825	HC	F	34.1	165.2	345	713	1059.5	1402	1693	1912	2117	2196				
GH2931	HC	M	32.2	156.2	344	616	922.7	1175	1365	1600	2050	2265	16 weeks	1742.50	1899.35	<0.001
GH2808	HC	M	25.9	180.1	417	696	1105.1	1308	1562	1904	2202	2279				
GH2848	HC	M	31.5	131.3	355	701	1013.4	1308	1563	1941	2131	2310				
GH2973	HC	M	36.2	179	380	642	976.5	1241	1542	1809	2165	2313				
GH3001	HC	M	35.3	144.3	410	748	1037.3	1301	1675	1917	2180	2348	18 weeks	1817.69	1997.14	<0.001
GH3042	HC	M	35.1	160.5	350	752	1075.7	1412	1669	1903	2223	2384				
GH2917	HC	M	32.5	175.9	401	533	1138.2	1400	1704	2003	2471	2576				
GH2872	HC	M	34.8	151.2	375	602	983.3	1445	1736	2035	2452	2638				

F: female, M: male.

**Table 3:** The ASVs showing different abundances between high- and high-market-weight chicken groups.

ASVs	Average Relative Abundance(%)		Enriched Groups	Phylum	Family	Genus
	LC	HC				
ASV20	1.99	3.28	HC	Firmicutes	Lachnospiraceae	*Ruminococcus_torques_group*
ASV482	0.15	0.36	HC	Firmicutes	Lachnospiraceae	*Lachnoclostridium*
ASV409	0.20	0.35	HC	Firmicutes	Lachnospiraceae	*Sellimonas*
ASV171	0.05	0.15	HC	Firmicutes	Lachnospiraceae	*Sellimonas*
ASV755	0.07	0.21	HC	Firmicutes	Lachnospiraceae	*unclassified_Lachnospiraceae*
ASV486	0.08	0.77	HC	Firmicutes	Ruminococcaceae	*Faecalibacterium*
ASV83	0.32	0.56	HC	Firmicutes	Ruminococcaceae	*Negativibacillus*
ASV412	0.06	0.10	HC	Firmicutes	Ruminococcaceae	*norank_Ruminococcaceae*
ASV100	0.33	0.71	HC	Firmicutes	Eubacterium_coprostanoligenes_group	*norank_Eubacterium_coprostanoligenes_group*
ASV51	0.06	0.20	HC	Firmicutes	Oscillospiraceae	*Oscillibacter*
ASV493	0.05	0.32	HC	Bacteroidota	Marinifilaceae	*Odoribacter*
ASV26	0.11	0.33	HC	Bacteroidota	Rikenellaceae	*Alistipes*
ASV169	0.05	0.28	HC	Bacteroidota	Rikenellaceae	*Alistipes*
ASV403	0.03	0.09	HC	Bacteroidota	Flavobacteriaceae	*norank_Flavobacteriaceae*
ASV5	6.33	2.91	LC	Firmicutes	Acidaminococcaceae	*Phascolarctobacterium*
ASV7	2.13	1.25	LC	Firmicutes	Acidaminococcaceae	*Phascolarctobacterium*
ASV17	0.66	0.04	LC	Firmicutes	Lactobacillaceae	*Lactobacillus*
ASV19	0.16	0.01	LC	Firmicutes	Lactobacillaceae	*Lactobacillus*
ASV101	0.13	0.05	LC	Firmicutes	Ruminococcaceae	*unclassified_Ruminococcaceae*
ASV487	0.10	0.02	LC	Firmicutes	Bacillaceae	*Bacillus*
ASV89	0.23	0.06	LC	Firmicutes	Lachnospiraceae	*unclassified_Lachnospiraceae*
ASV673	0.18	0.03	LC	Fusobacteriota	Fusobacteriaceae	*Fusobacterium*

**Table 4:** Annotation of metabolites enriched in high- and low-market-weight-chicken groups.

ID	Metabolites	*p* Value	VIP Value	Enrichment Group	ID	Metabolites	*p* Value	VIP Value	Enrichment Group
M374T30	N-arachidonoyl-l-alanine	0.001	1.32	HC	M335T31	Docosatrienoic acid	0.021	3.17	LC
M276T184	Luvangetin	0.003	1.71	HC	M230T22	Acetaminophen sulfate	0.021	2.52	LC
M357T532	Beta-estradiol 17-valerate	0.003	1.39	HC	M377T208	Riboflavin	0.019	3.51	LC
M429T49	(1-acetyloxy-3-hydroxy-6,8a-dimethyl-7-oxo-3-propan-2-yl-2,3a,4,8-tetrahydro-1h-azulen-4-yl) 4-hydroxybenzoate	0.004	2.70	HC	M339T24	Gly-His-Lys	0.019	2.24	LC
M173T114	Menadione	0.008	2.59	HC	M353T154	pGlu-Glu-Pro-amide	0.019	3.33	LC
M202T126	Pantothenic acid	0.008	2.54	HC	M291T59	Trimethoprim	0.017	1.86	LC
M227T180	Metyrapone	0.014	1.44	HC	M339T154	Cilastatin	0.017	3.87	LC
M393T31	Chenodeoxycholate	0.015	1.08	HC	M325T24	Hydroquinidine	0.017	2.10	LC
M233T60	Ile-Thr	0.017	1.14	HC	M311T25	Thymol-beta-d-glucoside	0.015	2.05	LC
M175T31	Alpha-ionone	0.023	1.64	HC	M259T28	1,3-benzenediol, 5-methyl-2-[(1r,6r)-3-methyl-6-(1-methylethenyl)-2-cyclohexen-1-yl]	0.014	1.65	LC
M273T255	Sempervirine	0.023	1.47	HC	M201T21	Bergaptol	0.014	2.75	LC
M249T20	D-myo-inositol-3,4,5,6-tetraphosphate	0.026	1.82	HC	M338T30	Erucamide	0.012	1.44	LC
M431T69	Eplerenone hydroxy acid	0.047	2.60	HC	M233T38	Riluzole	0.012	3.12	LC
M438T196	N-arachidonoyldopamine	0.047	1.25	LC	M242T134	Etodolac	0.010	2.87	LC
M285T157	3′,4′-dimethoxy-2′-hydroxychalcone	0.043	2.32	LC	M482T190_2	1-hexadecyl-sn-glycero-3-phosphocholine	0.008	2.14	LC
M503T239	Beclomethasone dipropionate	0.043	1.27	LC	M306T255	His-Lys	0.007	2.24	LC
M210T31	Phosphocreatine	0.043	2.29	LC	M297T25	Ostruthin	0.007	2.15	LC
M329T109	11beta-hydroxyprogesterone	0.035	1.99	LC	M162T24	5-hydroxy-3,4-dihydro-2(1h)-quinolinone	0.006	3.69	LC
M170T405	1-methylhistidine	0.032	1.88	LC	M290T99	Fingolimod	0.005	2.79	LC
M363T154	Glufosinate	0.032	4.22	LC	M294T306	Asn-Tyr	0.002	2.37	LC
M303T201	Hesperetin	0.032	2.07	LC	M312T344	5-hydroxydiclofenac	0.001	3.02	LC
M217T25	Chlorophene	0.029	2.61	LC	M249T287	2-aminoethylphosphonic acid	0.000	2.85	LC

## Data Availability

The datasets generated from this study have been submitted to the CNGB Sequence Archive (CNSA) of China National GeneBank DataBase (CNGBdb) with accession code CNP0003145.

## Supplementary Materials

The following supporting information can be downloaded at: https://www.mdpi.com/article/10.3390/ani13193034/s1, Table S1: Sequencing results of cecal microbiota from Guizhou yellow chickens; Table S2: Relative abundance of cecal microbiota at the phylum level; Table S3: The relative abundance of cecal microbiota at the genus level; Table S4: Co-abundance groups (CAGs) enriched in the high-market-weight chickens or low-market-weight chickens; Table S5: The overlap of differential bacteria groups at ASV, genus and CAG levels.
